# An analysis of community perceptions of mosquito-borne disease control and prevention in Sint Eustatius, Caribbean Netherlands

**DOI:** 10.1080/16549716.2017.1350394

**Published:** 2017-08-02

**Authors:** Teresa E. Leslie, Marianne Carson, Els van Coeverden, Kirsten De Klein, Marieta Braks, Anja Krumeich

**Affiliations:** ^a^ Eastern Caribbean Public Health Foundation, Sint Eustaitus, Caribbean Netherlands; ^b^ Pathobiology and Population Studies, Royal Veterinary College, London, UK; ^c^ Department of Social Sciences, Wageningen University, Netherlands; ^d^ RIVM, Bilthoven, Netherlands; ^e^ Health Medicine and Life Science, University of Maastricht, Maastricht, Netherlands

**Keywords:** Dengue, Chikungunya, Zika, community participation, prevention

## Abstract

**Background**: In the Caribbean, mosquito-borne diseases are a public health threat. In Sint Eustatius, dengue, Chikungunya and Zika are now endemic. To control and prevent mosquito-borne diseases, the Sint Eustatius Public Health Department relies on the community to assist with the control of Aedes aegypti mosquito. Unfortunately, community based interventions are not always simple, as community perceptions and responses shape actions and influence behavioural responses

**Objective**: The aim of this study was to determine how the Sint Eustatius population perceives the Aedes aegypti mosquito, mosquito-borne diseases and prevention and control measures and hypothesized that increased knowledge of the virus, vector, control and prevention should result in a lower AQ1 prevalence and incidence of mosquito-borne diseases.

**Methods**: This study was conducted in Sint Eustatius island in the Eastern Caribbean. We combined qualitative and quantitative designs. We conducted interviews and focus groups discussions among community member and health professional in 2013 and 2015. We also conducted cross-sectional survey to assess local knowledge on the vector, virus, and control and prevention.

**Results**: The population is knowledgeable; ©however, mosquito-borne diseases are not the highest health priority. While local knowledge is sometimes put into action, it happens on the 20 household/individual level as opposed to the community level. After the 2014 CHIK outbreak, there was an increase in knowledge about mosquito control and mosquito-borne diseases. Discussion: In the context of Sint Eustatius, when controlling the Aedes population it may be a strategic option to focus on the household level rather than the community and build collaborations with households by supporting them when they actively practice mosquito 25 control. To further increase the level of knowledge on the significance of mosquito-borne diseases, it may also be an option to contextualize the issue of the virus, vector, prevention and control into a broader context.

**Conclusion**: As evidenced by the increasing number of mosquito-borne diseases on the island, it appears that knowledge amongst the lay community may not be transferred into 30 action. This may be attributed to the perception of the Sint Eustatius populations that mosquitoes and the viruses they carry are not a high priority in comparison to other health concerns.

## Background

As evidenced by the increasing number of outbreaks in the Caribbean during the past decade, mosquito-borne diseases are a major public health concern [1,2]. As dengue, Chikungunya and Zika are now endemic, mosquito-borne diseases are a threat on the island of Sint Eustatius. To control and prevent mosquito-borne diseases, the Sint Eustatius Public Health Department relies on the community to assist with the control of *Aedes aegypti* mosquito [3,4].

Unfortunately, community based interventions are not always simple, as community perceptions and responses shape actions and influence behavioural responses [5,6]. Difficulty arises when community knowledge does not align with established strategies as this may inhibit prevention and control measures in the community [7–9]. Accordingly, it is critical to measure the level of community knowledge. Thus, the major hypothesis of this study is that high community knowledge of the vector, virus, control and prevention measures in the community result in lower prevalence and incidence of mosquito-borne diseases.

A dengue sero-survey conducted on Sint Eustatius between October and December 2011 revealed that dengue is endemic. Laboratory tests showed that 90.1% of the population had measurable past infections (antibodies against flaviruses) [10]. Between July and August 2012 a mosquito survey revealed that over 75% of the homes surveyed were positive for *Aedes aegypti*. Garbage related and domestic use containers contributed to 95% of the larvae sampled, while homes without door and window screens contributed to 82% of the sampled adults. The average number of female mosquitoes per household was five [11].

The Public Health Department is the local organization responsible for the prevention and control of mosquito-borne diseases, which are presented as a community issue with dual input from the Public Health Department and the general public [12–14]. The department conducts surveillance throughout the year and implements a community approach focusing on personal actions and responsibilities. To eliminate mosquitoes, biological (BTI and fish) and chemical (ABATE) controls are used. This programme has been in place for several years, with mosquito-borne diseases becoming an increasingly important component of the department’s work. Other activities include community outreach activities and information campaigns through TV, radio and pamphlets. Communication is focused on raising awareness of the vector, the viruses and control and prevention strategies. A jingle which is broadcast on the radio also spreads a message about the importance of cleaning your surroundings to prevent mosquito breeding.

Successful, long-term public health responses hinge on the behaviour of the local community [15–19]. After continuous communication, understanding the way the local population has come to conceptualize the vector, the viruses, and control and prevention activities may work to focus interventions. Here we report the results of a two-part cross-sectional study conducted between 2013–2015 involving semi-structured interviews (2013 & 2015) and a survey (2015) aimed at measuring local knowledge. The study was conducted in two phases between 2013–2015. The first (2013) phase was a qualitative study measuring dengue health behaviour, as dengue was the only mosquito-borne disease known to be circulating. The second phase was conducted in 2015, after the 2014 Chikungunya outbreak, and included qualitative and quantitative component focusing on knowledge surrounding mosquito-borne diseases generally. A specific point of interest was if and how knowledge increased/changed after the 2014 Chikungunya outbreak.

## Methods

### Study area and study population

Sint Eustatius is one of the smallest islands in the Eastern Caribbean. Located in the northern Leeward Islands, it has an area of 21 square kilometers (8.1 sq mi), a population of about 3200 inhabitants, and is a special municipality of the Kingdom of the Netherlands. English is predominately spoken and written [20,21]. All study participants were legal adults aged 18 or above, residents, and able to speak and understand English. No individuals were duplicated in any of the studies.

## Study design

### Part 1: qualitative study 2013

Thirteen individual, semi-structured interviews and two focus groups were conducted within the resident community of Sint Eustatius between May to July 2013 by TC and MC (Table 1). Both lay respondents and health professionals were included in the individual interviews. Convenience and snowball sampling provided access to the population of Sint Eustatius with the Public Health Department assisting in the recruitment of participants. Participants were identified through the use of formal and informal networks as well as by the primary author (TL). Each focus group contained participants from a shared workplace who were familiar with other participants in their group [22–24].

**Table 1.** Demographics of sample population from the two-part cross-sectional study conducted between 2013–2015 involving semi-structured, individual and focus group interviews (2013 & 2015) and survey (2015).

		Total # (M/F)		Age range	
Interview type		2013	2015	2013	2015
Individual	Laypersons	10 (6/4)	24 (12/12)	20–70	24–60
	Health professionals	3 (1/2)	0	40–60	-
Focus group	Laypersons, Group 1	6 (2/4)	0	20–60	-
	Laypersons, Group 2	4 (1/3)	0	20–60	
Survey	Laypersons	0	60 (24/36)	-	19–60
Total (qual.)		24	24		
Total (quan.)		0	60		
Total		24	84		

The content of the questions surrounded perceptions and knowledge of mosquitoes, the viruses (severity, danger, etc.), and control and prevention (methods, responsibility, effectiveness, etc.). It was assumed that the interviewed health professionals had a higher awareness of mosquito-borne diseases than the lay population and questions were tailored to account for this (TL, AK, MC). Consultations and debriefings were carried out weekly (TL, MC) during the data collection phase.

### Part 2: qualitative and quantitative study 2015

#### Qualitative study

From October–December 2015 a second study was carried out amongst the lay community (TL, KK and EvC) following the 2014 Chikungunya outbreak to determine if perceptions of the vector, virus and prevention and control had increased. With the assistance of the public health department, 24 participants were recruited from the general population of Sint Eustatius using convenience and snowball sampling (Table 1). Participants were identified through the use of formal and informal networks as well as by the primary author (TL) and semi-structured interviews were conducted. During the data collection phase consultations and debriefings were carried out weekly (TL, KK, EcV). As in the 2013 study, the general themes of the virus, the vector and control and prevention were formulated prior to data collection.

#### Quantitative survey

To triangulate the findings from the 2013 and 2015 qualitative studies, a cross sectional quantitative survey was conducted (TL, KK and EcV) to obtain information on local knowledge on the vector, virus and control and prevention. Sixty individuals who did not participate in the 2013 or 2015 qualitative surveys were recruited (Table 1). Recruitment procedures followed the 2013 and 2015 qualitative studies.

## Analytical approach

### Part 1: qualitative study 2013

Interviews were prepared for analysis by being transcribed into Microsoft Word shortly after data collection (MC). Thematic data analysis was then conducted as follows: interview transcripts were read twice and then analyzed using the Cut and Sort processing technique as described by Stewart et al. [25] and Ryan and Bernard [26]. Sections in the texts relevant to the problem statement and objectives were identified. Data were coded with the predetermined themes (virus, vector, and control and prevention). Data that could not be coded were identified and analyzed later to determine if they represented a new theme or a subtheme. Sorted materials were then compiled and examined for variations within these themes (TL & MC). Transcripts were coded and sorted in Microsoft Excel.

### Part 2a: qualitative study 2015

Interviews were prepared for analysis by being transcribed into Microsoft Word shortly after data collection (KK and EvC). All methods used in 2013 were duplicated in 2015.

### Part 2b: quantitative survey 2015

Data from the quantitative survey were compiled and summarized using Microsoft Excel Software (KK and EvC) (Tables 3 and 4).

**Table 2.** Themes and subthemes identified during Part 1: 2013 qualitative study and Part 2a: 2015 qualitative study investigating dengue and mosquito-borne disease knowledge and perceptions amongst the lay community on Sint Eustatius.

Theme/Year	Part 1: 2013	Part 2a: 2015
Mosquito	Non-species specific	Non-species specific
	Nuisance	Nuisance
	Normal to have mosquitoes	Normal to have mosquitoes
	Cleanliness	Cleanliness
	- Water	- *Roaminganimals*
	- Vegetation	*- Other insects*
		- Vegetation (coralita)
Prevention and control	Community/government alliance	Community/government alliance
	Household level as opposed to community	Household level as opposed to community
	Government/fogging	Government/fogging
Disease	A concern - increasing	A concern during outbreak
	Increased concern during outbreak	Increased concern during outbreak
	Flu-like	Flu-like
		Differentiation with flu
		Negative economic influence on island
		- Tourism
		- Labour market

Note: Underlined and italicized subthemes represent themes that were at first identified as outlier themes which were later seen as related to the cleanliness sub-theme on an island-wide level.

**Table 3.** Knowledge and perceptions of risk related to mosquito-borne diseases amongst 60 lay participants, identified during Part 2b: quantitative survey on Sint Eustatius.

	Number (%)
**Had a mosquito-borne disease**	
Yes	15 (25)
I am not sure	7 (11.7)
No	38 (68.3)
**Family members that had a mosquito-borne disease**	
Yes	30 (50)
I am not sure	13 (21.7)
No	17 (28.3)
**Perceived risk**	
Not at risk at all	2 (3.3)
Slightly at risk	18 (30.1)
Very at risk	31 (51.7)
Extremely at risk	9 (15)
**Perceived necessity of control measures**	
Extremely necessary	28 (46.7)
Very necessary	19 (31.7)
Slightly necessary	8 (13.3)
Very unnecessary	1 (1.7)
Not necessary at all	4 (6.7)

**Table 4.** Knowledge about mosquito-borne disease transmission, mosquito-breeding sites and prevention methods amongst 60 lay participants identified during Part 2b: quantitative survey on Sint Eustatius.

Variable	Number (%)
**Mode of disease transmission**	
Mosquito bites	57 (95)
Breathing bad air	3 (5)
Contact with a person that has a mosquito-borne disease	13 (21.7)
Exposure to rain	6 (10)
Bad smell	1 (1.7)
**Mosquito-breeding sites**	
Stagnant water	51 (85)
Running water	2 (3.3)
Dirty places	38 (63.3)
Tires	44 (73.3)
Food products	5 (8.3)
Garden	33 (55)
Indoors	15 (25)
Other namely*	2 (3.3)
**Sources of information**	
I did not search for information	11 (18.3)
Newspaper	23 (38.3)
Leaflets	20 (33.3)
Television	25 (41.7)
Internet	31 (51.7)
Family/friends	21 (35)
Health-care personnel	14 (23.3)
Public Health Department	34 (56.7)
Other namely*	2 (3.3)

### Ethical approval and consent

The study protocol was approved by the University of Maryland Institutional Review Board (11–0059) and The Caribbean Public Health Agency Research Ethics Committee (00010238). All participants provided informed consent to participate in the study.

## Results

### Part 1: qualitative study

#### Perception of the mosquito

In 2013, participants did not differentiate between mosquito species. Awareness of which mosquito species transmits dengue and how it can be identified was variable and concern for preventing mosquito breeding and bites was found to be non-species specific. People were very aware that the mosquito breeds in stagnant water in containers around the home. Mosquitoes and being bitten were viewed as an inescapable part of everyday life. Although mosquitoes were seen as something that is part of life, many people felt that mosquitoes were irritating. Mosquitoes were generally perceived as negative beyond concerns about health or infection.

In 2013 the theme of cleanliness was interlinked with concerns surrounding mosquitoes and the degree of potential mosquito breeding sites around homes. Risk of dengue appeared to be measured by the degree of ‘cleanliness’ of a person’s yard, with a clean yard being characterized by factors such as a lack of standing water, garbage and unattended plants. Some participants provided explanations for high mosquito counts and its relation to cleanliness (Table 2).

#### Perception of control and prevention

The Public Health Department’s focus on mosquito population control was most often perceived as positive and most people were willing to assist on a household level. Keeping a yard clean appeared to be important; however, the degree to which this responsibility extended beyond one’s own property was variable. Although keeping water containers, garbage and other water catchments appeared to be associated with mosquitoes and potential disease, neighbours talking to neighbours about these issues was not common practice.

There was variation among participants about the work of the vector controllers. While some agreed that vector controllers need community assistance, others did not notice their presence.

Some participants looked to the past to identify the best practices for mosquito control. Individuals who viewed the Health Department as doing very little to control mosquitoes often defined fogging as the best method of control. Spraying/fogging was positively remembered by those who had lived long on the island as something conducted by the government in the past. The view that mosquito control should lie primarily in the hands of the government appeared to be linked to the view of spraying or ‘fogging’ of the island as the most appropriate approach (Table 2).

#### Perception of disease

In 2013 dengue was the only known mosquito-borne disease on the island and, thus, the only disease associated with mosquitoes on Sint Eustatius. The most important lay community health concerns were focused on non-communicable and lifestyle associated diseases such as diabetes, hypertension, obesity and cancer. These were also considered the most dangerous. Among infectious diseases, AIDS was considered the most dangerous. The health professional’s perspective on the island’s dengue situation in 2013 differed. While Public Health professionals identified dengue as a serious concern, they were also aware that the community generally did not define dengue as a major public health threat. Data reveals that the immediate danger of mosquito-borne diseases in the community seemed to be low. However, some expressed an increasing concern. In 2013 people described that dengue may be an issue if there were an outbreak and when friends and/or family members contracted the disease (Table 2).

### Part 2a: qualitative study

**Figure 1.**
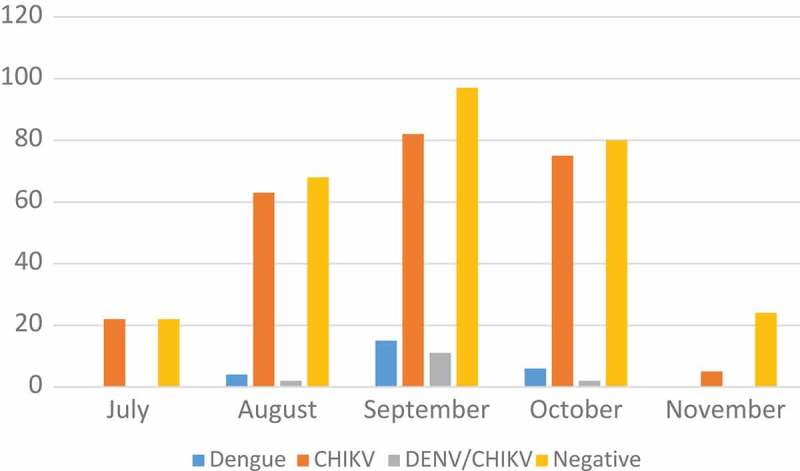
2014 Chikungunya outbreak in Sint Eustatius, Caribbean Netherlands (number of suspected cases by month). Note: Suspected cases include Dengue, CHIKV and CHIKV/DENV coinfection.

The 2014 Chikungunya outbreak indicates how quickly vector-borne diseases can spread on Sint Eustatius. Between July–December there were 268 suspected cases of chikungunya-like febrile illness. Twenty-three of 268 (9%) patients tested via ELISA were confirmed to have had recent DENV infection, while 172 of 234 (74%) patients tested by RT-PCR were confirmed to have chikungunya. Fourteen patients were shown to be co-infected with both Chikungunya virus (CHIKV) and dengue virus (DENV). There were no deaths or reports of severe disease associated with the outbreak (Figure 1). This section presents the beliefs and knowledge of people after this 2014 Chikungunya outbreak.

#### Perception of the mosquito

Similar to 2013, participants continued to discuss mosquitoes in a general way and did not differentiate between mosquito species. When discussing mosquitoes there was no mention of *Aedes aegypti*. Mosquitoes were defined again as a general nuisance and were part of living in the Caribbean.

In 2015 cleanliness continued to remain a sub-theme. However, participants were more specific about what they defined as clean. Often, coralita was specifically mentioned as a plant that contaminates yards and may be responsible for harbouring mosquito-breeding sites. Coralita (*Antigonon leptopus*) is an invasive plant on Sint Eustatius and commonly defined as destructive as it covers a large percentage of the island and potentially inhibits people’s ability to see if there are mosquito-breeding sites underneath. When discussing mosquitoes, in addition to coralita, other nuisances were also mentioned including roaming livestock (cows, goats, sheep, donkeys) and other insects (flies, roaches, rats, etc). Often, these roaming animals and other insects were identified as more bothersome than mosquitoes (Table 2).

#### Prevention and control

In 2015 prevention of mosquito-borne diseases was linked with vector control activities.

While not mentioning vector control, ‘doing something about mosquitoes’ falls within the jurisdiction of vector control. Other individuals spoke directly about the government programmes. Similar to 2013, individuals continued to discuss the past fogging activities of the Public Health Department as the best method of control. Another participant mentioned how the Public Health Department used to provide fish in the past, as a way to control mosquitoes – a practice that was described as not existing any more. However, the Public Health Department does utilize fish as a biological control method upon request.

In 2015 many individuals directly discussed what they do to prevent mosquitoes on a household level. For mosquito control inside the house the use of chemical insect repellents, collectively termed ‘flit’ or using bug spray were seen an obvious solution where ‘flitting’ offered household protection. Although recommended by the Public Health Department, the use of bed nets was not seen as an applicable method since bed nets cannot be purchased on the island. Window screens were seen as partially acceptable and available, but at a high cost. Since the installation of screens is costly, screens are seen mostly in newer homes. However, screens were also not seen as necessary or a priority to have in homes if ‘flit’ or fans were used. Using ‘flit’ or turning on a fan to blow the mosquitoes away was perceived as a cheaper option. People also utilize air-conditioning; however, this was mainly associated with comfort levels and personal preference rather than a health concern (Table 2).

#### Perception of disease

Similar to 2013, mosquito-borne diseases were not discussed as a primary threat. However, during periods of outbreak, the level of concern rises and then subsides afterwards. Some described that only when an individual has experienced a mosquito-borne infection would they actually realize how critical it is to prevent and control the mosquito. However, participants did note that when people contracted a disease they recovered after several days. The rapid recovery of infected individuals may influence the prevention measures taken by the general population as they do not view the disease as a serious health risk. Also, some discerned an association between dengue and the flu. Similar to the flu, upon an initial sickness, in time the individual recovers. Unlike 2013 some participants did in fact differentiate between the flu and mosquito-borne diseases.

One topic discussed in 2015 and not discussed in 2013 was the larger economic impacts of mosquito-borne diseases. The main sector that was seen as impacted by mosquito-borne diseases was the tourism sector. It was believed that if the island harbors too many mosquitoes and the viruses they carry, tourists may select other destinations for vacation purposes (Table 2).

### Quantitative results

#### Perception of the mosquito

Similar to the qualitative study, the quantitative results (Table 3) illustrated that a high percentage of the sample population (95.0%) knew that dengue and chikungunya are transmitted via mosquito bites. Only two respondents selected the *other, namely-option*. One respondent wrote down ‘abandoned cisterns’, while another wrote down a list with several options, namely: dirty surroundings; garbage that is not tended to; dirty water around your home; the weather; heat. While not mentioning mosquitoes, these individuals highlighted places where mosquitoes can breed (Table 3).

#### Perception of control and prevention

To assess the perceived responsibility for implementing mosquito control, participants were asked to scale how responsible they thought different actors were in mosquito control, from 0% (not responsible at all) to 100% (very responsible). Participants perceive that the responsibility for implementation of mosquito control lies first with the households (77.2%), second with the Sint Eustatius government (75.5%), and third with the Caribbean regional government (67.6%). The Dutch government scored lowest (65.9%). These results suggest that there is consensus among the community that mosquito control and disease prevention should be a combined effort between the Public Health Department and households when trying to control mosquitoes and prevent mosquito-borne diseases.

#### Perception of disease

Survey results regarding the level of perceived urgency surrounding mosquito-borne diseases are provided in Table 4. Most of the study population, 63.3%, indicated never to have had a mosquito-borne disease. Of all the respondents, 50.0% knew a family member that had a mosquito-borne disease. In all, 51.7% of the respondents felt at risk for acquiring a mosquito-borne disease. Almost half of the respondents (46.7%) felt that control measures were extremely necessary.

## Discussion

In addition to dengue, Chikungunya and Zika are endemic in Sint Eustatius and it is likely that other mosquito-borne diseases such as Yellow Fever (YF) will re-emerge. Unlike YF, there is no vaccine available for most mosquito-borne diseases and prevention rests on the delicate balance of state mandated activities and the engagement of communities. However, engaging communities is not simple as community perspectives are rarely homogenous. Incorporating variations and similarities in perceptions are valuable for effective prevention and control [27].

One theme that continuously arose in both 2013 and 2015 was the concept of cleanliness. One message that is promoted by the Public Health Department is that a ‘clean’ yard will reduce the risk of mosquito-borne diseases. A clean yard was primarily described as one that did not have standing water, garbage and excess bushes. This concept of a ‘clean’ yard may be attributed to the Department’s jingle on the radio which states ‘keep your surroundings clean, turn your buckets upside down, reduce dengue fever all around.’ Cleanliness is often a common theme found in studies surrounding knowledge of mosquito-borne diseases. A 2017 study from Belagavi city, India, Kularni et al. [28]. found that 36.6% considered drainage and garbage as a common breeding place. In a 2013 study of four provinces (Kabar Danga, Indaragora and Kenduadihi) of Bankura Town, India, identified polluted water of drains as a major source of mosquito-breeding followed by garbage [29].

While in 2013 cleanliness was discussed in a more general context, in the 2015 qualitative survey cleanliness was expanded to include the greater environment with the invasive plant coralita being specifically mentioned as a culprit for mosquito breeding. Roaming animals (cows, goats and sheep) and other insect pests (roaches and flies) were also identified. All of these factors can actually result in increased mosquito-breeding sites. In Sint Eustatius, roaming animals are notorious for knocking over garbage cans in the night, and while people generally try to clean up the garbage that the animals have spread around, some is overlooked. Items like Styrofoam food containers, cups and food cans may end up in the bushes and coralita can conceal them. When it rains they can become perfect breeding grounds for mosquitoes. Depending on the trash items spread by the roaming animals, other insect pests may also become a problem.

This differs from similar research in a costal rural area of India where Zaidi et al. [30]. found that a significant number of people had no knowledge about the breeding sites of mosquitoes. More than one-third of the interviewees did not know of any preventive measures against mosquitoes at the household and community level [30]. In Sint Eustatius, the participants seem to be making a connection between environmental and waste management concerns and its actual relation to mosquito-breeding sites [31]. These concerns may impact the economy as high mosquito counts and endemic mosquito-borne diseases may dissuade tourists from visiting the island. It is possible that by bringing these important island issues into the discussion of mosquito-borne disease control and prevention, the Public Health Department may be able to build stronger interventions.

While cleanliness was a constant theme, there were several knowledge gaps identified in this study. Although mosquito-borne diseases were generally perceived as a relatively recent issue, in 2013 and 2015 mosquitoes were not. While, some people had direct or indirect experiences with mosquito-borne diseases, in 2013 and 2015 everyone had daily experiences with mosquitoes and bites, irrespective of if it was recognized as caused by *Aedes aegypti*. In both years, mosquitoes were discussed very generally with no specific reference to *Aedes aegypti*. Furthermore, the view of mosquitoes as primarily a societal nuisance appears to be prevalent and sometimes often overshadowed the vector’s role as a transmitter of potentially deadly diseases. Nevertheless, the quantitative data show that 95% of the sample population identifies mosquitoes as the main source of infection. This result differs from a study in an Indian urban locality where 99% of respondents from an urban area in India did not know the mode of transmission for dengue [32].

In Sint Eustatius taking preventative measures around and within one’s home was not a novel concept associated simply with avoiding disease, but rather a long-standing tradition for a community living in a tropical context. Thus, the use of bug spray (flit), screens, mosquito rackets and so on contribute to a more comfortable life and are solutions which are relatively accessible. This community focus on the everyday dislike of mosquitoes over any perceived health risks was also identified by Phuanukoonnon et al. [33] in dengue-endemic Northern Thailand. In this study participants focused on the inconvenience and annoyance of mosquitoes rather than the diseases transmitted.

In 2013, chronic diseases were identified as major health concerns on the island, and, in contrast to a recent sero-survey [10] where 90% of the population was characterized as being exposed to one or more dengue serotypes, many participants did not recall having a mosquito-borne disease. In the 2015 quantitative survey, 69% also describe themselves as never having a mosquito-borne disease. Self description of never having had a mosquito-borne disease may result from many individuals experiencing mild/moderate symptoms or no symptoms, which is characteristic of both dengue and Chikungunya [34]. Often symptoms are flu-like and may often be misdiagnosed, further influencing the perceived risk level. In the quantitative component of the 2015 study most individuals described how they were slightly at risk (18; 30.1%) to very at risk (31; 51.7%). This may indicate that while there is concern, many continue not to see mosquito-borne diseases as high risk.

While the perceived risk of disease may be low, in 2015 the majority of people described prevention and control efforts as extremely necessary (46.7%) and very necessary (31.7%). The significance of prevention and control may be attributed to the 2014 Chikungunya outbreak resulting in the Sint Eustatius population actually experiencing a mosquito-borne disease. In Puerto Rico, Perez et al. [35,36] found that residents considered dengue an issue within the community mainly when people were aware of cases within the population.

Participants perceived that the responsibility for implementation of mosquito control lies with households (77.5%) and the Sint Eustatius government (75.5%). Furthermore, the 2015 quantitative study shows that 56.7% of the sample population identified the Public Health Department as the main organization providing information on mosquito control and mosquito-borne disease prevention (Table 4). This lies in contrast to a 2003 study in an urban locality in India where the overwhelming majority of respondents (56.8%) were of the attitude that mosquito control was the government’s responsibility and very few (8%) responded that it was the responsibility of the community [32].

While the Public Health Department has conceptualized its approach to mosquito-borne diseases as a community issue requiring a united front, in both 2013 and 2015 the approach to prevention and control appears to be divided between the actions of individuals and the actions of public health professionals. While the quantitative study illustrates that individuals identify the community and government as equal partners in mosquito control, the qualitative data reveal that there seems to be a lack of community ownership of the Public Health Department’s activities and practices beyond what can be done within the confines of one’s own home. Although according to the Public Health Department mosquito-borne diseases are described as an island-wide issue, within the community, individual actions and individual precautionary measures taken, struggles in reality to transverse the neighbour’s fence. This behaviour suggests that the concept of a community approach to mosquito control is, in fact, not a reality. Perez-Guerra et al. [35] also found this behavioural characteristic in Puerto Rico where individuals often blamed their neighbours for not contributing appropriately and neglecting the combined efforts necessary for successful community based programmes. In Sint Eustatius, it may be a strategic option for the Public Health Department to focus on the household level rather than the community and build collaborations with households by supporting them when they actively practise mosquito control. In this way, through the household, the Public Health Department can work to build community awareness.

While the Public Health Department focuses on the elimination of mosquito-breeding sites, in both 2013 and 2015 some participants preferred government to have a greater role. In 2015 more individuals reported seeing vector controllers conducting household surveys. One even mentioned that they told people about how they could get BTI larvicides at the Public Health Department. However, others reported that they thought the government could do more. Some people may not see vector controllers working, as vector controllers work in the mornings when many are working. This is similar to a study by Ghosh et al. [29] where there was a disparity regarding the visibility and usefulness of government efforts. About 28% of the sample mentioned that they were aware of government efforts (cleaning garbage, drains, the spraying of chemicals and larvicides) and about 38% did not know. About 34% mentioned that government effort is lacking [29].

In Sint Eustatius the preferred role was that government periodically fog the island. Compared to highly visible interventions such as fogging, house visitations may not be perceived as an equally and obvious active tactic to the community. This may raise concerns, from a lay perspective, if mosquitoes and mosquito-borne diseases are an on-going priority for the government. This is similar to a 2003 [32] study from an urban area in India where the majority of people identified the use of chemicals as the best approach to control mosquitoes (42.9%) [32].

There are specific reasons why the Sint Eustatius Public Health Department does not rely on fogging as a means of vector control. Permethrin is used as the primary chemical when fogging and is the only chemical on the market that can be legally used. Continuous fogging would ultimately result in mosquitoes becoming resistant and fogging becoming ineffective in the long term [37,38]. Furthermore, fogging can cause damage to the local fauna on the island. For example, Sint Eustatius is home to *Athrophora eustatiensis*, its very own native bee and permethrin is extremely toxic to bees [39,40]. Severe losses may be expected if bees are present at treatment time. Thus, while fogging would provide an active and visible role of the Public Health Department, it is only used during periods when there is an outbreak to take down those mosquitoes who may be carrying virus. More effective communication is needed to inform the population about why vector control focuses on mosquito-breeding sites and why fogging is not the preferred method.

## Study limitations

While this study provides interesting results, sampling does introduce a limitation as it was not random and participants were identified via snowball and convenience sampling. Therefore, representativeness of the sample is not guaranteed and subjects the study to self-selection bias. Given this limitation, the study does provide some novel results that may improve the efforts of the Public Health Department on Sint Eustatius. While the results are specific to Sint Eustatius and cannot be extrapolated to other regions, similar studies are proving important when working to engage communities in the control and prevention of mosquito-borne diseases.

## Conclusion

The Sint Eustatius population is somewhat knowledgeable about the virus, the vector and prevention and control measures. It seems as if knowledge slightly increased after the 2014 Chikungunya outbreak. Given this knowledge, as illustrated by the 2014 Chikungunya outbreak, when introduced on the island, CHIKV spread quickly. Thus, knowledge did not result in low infection rates. One possible explanation for this is that knowledge is not always being translated into community action. Community inaction may be related to the fact that, except for periods of outbreak, people do not see mosquito-borne diseases as a primary concern. However, inaction could also be related to the fact that many of the actions are personal and do not translate over to the community. This may represent the failure of the Public Health Department’s ability to utilize community networks. The reasons why on such a small island there is very little cooperation within communities, and between the Public Health Department and the community at large, requires further investigation.

## Paper context

This paper explores local knowledge surrounding mosquito borne diseases on the small Caribbean island of Sint Eustatius. Three areas are explored including local knowledge on the vector, viruses, and control and prevention practices. It appears that knowledge amongst the lay community may not be transferred into action. This may be attributed to the perception of the Sint Eustatius populations that mosquitoes and the viruses they carry are not a high priority in comparison to other health concerns. Documenting community knowledge should assist the Public Health Department with designing strategies that target knowledge gaps and ultimately work to improve control and prevention efforts.
